# A Polymorphism (rs2295080) in *mTOR* Promoter Region and Its Association with Gastric Cancer in a Chinese Population

**DOI:** 10.1371/journal.pone.0060080

**Published:** 2013-03-29

**Authors:** Ming Xu, Guoquan Tao, Meiyun Kang, Yan Gao, Haixia Zhu, Weida Gong, Meilin Wang, Dongmei Wu, Zhengdong Zhang, Qinghong Zhao

**Affiliations:** 1 Department of Environmental Genomics, Jiangsu Key Laboratory of Cancer Biomarkers, Prevention and Treatment, Cancer Center, Nanjing Medical University, Nanjing, China; 2 Department of Genetic Toxicology, The Key Laboratory of Modern Toxicology of Ministry of Education, School of Public Health, Nanjing Medical University, Nanjing, China; 3 Department of General Surgery, Huai-An First People’s Hospital Affiliated to Nanjing Medical University, Huai-an, China; 4 Core Laboratory of Nantong Tumor Hospital, Nantong, China; 5 Department of General Surgery, Yixing Cancer Hospital, Yixing, China; 6 Department of General Surgery, The Second Affiliated Hospital of Nanjing Medical University, Nanjing, China; IFOM, Fondazione Istituto FIRC di Oncologia Molecolare, Italy

## Abstract

**Background:**

As an imperative part of PI3K/Akt/mTOR pathway, mammalian target of rapamycin (mTOR) has been demonstrated to increase in gastric cancer cells and tumors. Our research explored the relationship between single nucleotide polymorphism (SNP) rs2295080 in *mTOR* promoter region and the risk of gastric cancer (GC).

**Methods:**

Seven hundred and fifty-three (753) gastric adenocarcinoma patients and 854 matched healthy subjects were recruited in the cancer association study and 60 tissues were used to test the expression of *mTOR*. Unconditional logistic regression was selected to evaluate the association between the rs2295080 T>G polymorphism and GC risk. We then examined the functionality of this promoter genetic variant by luciferase assay and EMSA.

**Results:**

Individuals with G allele had a 23% decreased risk of GC, comparing with those carrying T allele (adjusted OR = 0.77, 95% CI = 0.65–0.92). This protective effect of G allele stood out better in male group. Meanwhile, GC patients carrying TG/GG genotype also displayed a decreased mRNA level of *mTOR* (*P* = 0.004). In luciferase assay, T allele tended to enhance the transcriptional activity of *mTOR* with an approximate 0.5-fold over G allele. Furthermore, EMSA tests explained that different alleles of rs2295080 displayed different affinities to some transcriptional factor.

**Conclusion:**

The *mTOR* promoter polymorphism rs2295080 was significantly associated with GC risk. This SNP, which effectively influenced the expression of *mTOR*, may be a new biomarker of early diagnosis of gastric cancer and a suitable indicator of utilizing mTOR inhibitor for treatment of GC.

## Introduction

Gastric cancer (GC) is the most frequently occurring cancer worldwide among men with 640,556 and women with 349,042 new cases and 738,069 related deaths in 2008 [1]. In consideration of the therapeutic efficiency, surgical resection can be primary curative treatment for earlier stage of GC patients [2]. Unfortunately, most gastric cancer patients are detected in advanced stage, during which period the tumor are unresectable any more. Furthermore, relapse after surgery is another terrible event for a poor 5-year survival rate. Considering the patients with advanced or recurrent gastric cancer, it is no doubt that discovery of biomarkers and their application accompanied with traditional diagnosis might be a valuable indication and an extensive help to formulate the prevention and treatment strategy.

Mammalian target of rapamycin (mTOR) consists of 2,549 amino acids arranged in highly conserved domain [3]. Structurally, mTOR contains a rapamycin-binding domain in its central part and a kinase domain in the C-terminus [4]. As a key component of PI3K/Akt/mTOR pathway, mTOR is closely relevant to capital cellular processes such as cell growth, proliferation, metabolism, migration, angiogenesis, and apoptosis [5]–[7]. Until now, *mTOR* products are concerned as two distinct complexes (i.e., mTORC1 and mTORC2) with different sensitivity [8], [9]. mTORC1 is a rapamycin-sensitive complex which includes mTOR plus FK506-binding protein 12 kDa (FKBP12), mammalian LST8 (mLST8), and the regulatory-associated protein of mTOR (raptor). In contrast, mTORC2 consists with Sin1, mLST8, and rapamycin-insensitive companion of mTOR (rictor) [10]–[12]. Dysregulation of *mTOR* often appears in various kinds of cancers during the carcinogenesis and deterioration. However, the reasons for this aberrant phenomenon are still glutted with debates.

A number of studies have investigated the role of single nucleotide polymorphisms (SNPs) of *mTOR* gene in the etiology of cancers in various organs, including esophageal cancer [13], lung cancer [14], bladder cancer [15], colon cancer, rectal cancer [16], and acute lymphoblastic leukemia [17]. Most of these SNPs locate in exons or introns with unknown functional effects. Recently, an increasing number of studies have focused on the SNPs seating in gene promoter region, which are proved to influence the binding ability with some transcriptional factors (TFs) and impact the following gene transcription. In this study, we hypothesized that *mTOR* rs2295080 T>G polymorphism within the promoter region might influence the susceptibility to GC. To test this hypothesis, we genotyped the frequency of *mTOR* rs2295080 to test its importance on GC risk in our ongoing, hospital-based, case-control study in a Chinese population. Consequently, we detected the *mTOR* mRNA levels with different genotypes in tissues of gastric cancer patients. Furthermore, we further characterized the functionality of this genetic variant on the *mTOR* promoter transcription by luciferase assay and EMSA.

## Materials and Methods

### Study Subjects

This study comprised 753 patients with histologically confirmed gastric adenocarcinoma and 854 cancer-free controls. All patients were recruited from the Cancer Clinical Research Base of Nanjing Medical University between March 2006 and January 2010. And all demographic and clinical information, including age, sex, tumor size, tumor site, histological types, depth of invasion, lymph node metastasis, distant metastasis, and TNM stage, were obtained using a short questionnaire and clinical medical records.

The frequency-matched controls to these cases by age (±5 years) and sex were collected at the same period and regions of unrelated genetic relationship with gastric disease and digestive system tumors. Each participant signed a written informed consent and donated 5 ml venous blood for genomic DNA extraction. The research protocol was approved by the institutional review board of Nanjing Medical University.

### DNA Extraction and SNP Genotyping

Genomic DNA was extracted from blood samples as described previously [18]. Genotyping was completed by TaqMan SNP Genotyping Assay using ABI 7900HT real-time PCR System (Applied Biosystems, Foster City, CA, USA) and Sequence Detection System version 2.4 (SDS 2.4). The sequences of primers and probes were available on request, and controls were contained for each plate to ensure accuracy of genotyping. The genotyping assay was performed by two persons independently in a blind fashion. More than 10% of the samples were randomly selected for confirmation, and the results were 100% concordant. Primers and probes were listed in Table S1.

### Cell Culture and Construction of Promoter Reporter Plasmids

One normal gastric mucosa epithelial cell line (GES-1) and three different gastric cancer cell lines (BGC-823, MGC-803, and SGC-7901) were adopted into this study. All cells were cultured in Dulbecco’s Modified Egale Medium/High glucose culture medium with 10% FBS, 10 mM HEPES, 2 mM L-glutamine, 1 mM pyruvate sodium, 100 U/ml penicillin, and 100 µg/ml streptomycin at 37°C in a humidified atmosphere containing 95% air and 5% CO_2_. Most of reagents were obtained from GIBCO (Burlington, Ontario, Canada).

In luciferase reporter plasmids, the human *mTOR* promoter sequences with different alleles for rs2295080 T>G polymorphism were synthesized and constructed into pGL3-basic vector (Promega, Madison, WI, USA) by Generay Company (Shanghai, China). All plasmids were confirmed by DNA sequencing. Primers involved in the test were listed in Table S2.

### Transient Transfection and Luciferase Assay

GES-1, BGC-823, MGC-803, and SGC-7901 cells were transfected by Lipofectamine 2000 (Invitrogen, Carlsbad, CA, USA) with 0.8 µg of each constructed vector, either with T allele or with G allele. Simultaneously, 10 ng pRL-SV40 per well was also transfected as an internal control for correcting transfection efficiency. Before it, cells were seeded on 24-well plates over night to ensure 90%–95% confluence at the time of transfection. Twenty-four hours after transfection, luciferase activity was measured by the Dual-Luciferase Reporter Assay System (Promega, Madison, WI, USA) and expressed as the ratio of Firefly luciferase to Renilla luciferase activities. All cells were done in triplicate with the same conditions.

### Electrophoretic Mobility Shift Assay (EMSA)

The sense probe sequences were as follows: rs2295080 T probe, 5′-AGGGTTCCCATCCCTGAGGAC-3′; rs2295080 G probe, 5′-AGGGTTCCCAGCCCTGAGGAC-3′. Nuclear proteins were extracted with NE-PERTM Nuclear and Cytoplasmic Extraction Reagents (Pierce, Rock-ford, IL, USA). DNA probes were prepared with the Biotin 3′-End DNA Labeling Kit (Pierce, Rock-ford, IL, USA). Electrophoretic mobility shift assay (EMSA) was performed with a LightShift Chemiluminescent EMSA Kit (Pierce, Rock-ford, IL, USA). Binding reactions were performed as follows: nuclear extracts (8 µg protein) and the 1× binding buffer with 2.5% glycerol, 5 mM MgCl_2_, 50 ng/µl poly (dI-dC), 0.05% NP-40, and 20 fmol biotin-labeled rs2295080 T/rs2295080 G probes were incubated on ice for 30 min in a volume of 20 µl. For competition studies, nuclear extracts were incubated with unlabeled oligonucleotide for 30 min before the addition of labeled oligonucleotide.

#### Expression levels of *mTOR* mRNA

Total RNA from 60 gastric cancer tissues, included in 753 cases, with different genotypes were extracted using Trizol Reagent (Invitrogen, Carlsbad, CA, USA). The mRNA was measured by quantitative real-time PCR (ABI 7300) after reverse transcription. GAPDH was used as an internal quantitative control for each sample. The primers used for mTOR amplification were F: 5′-TTGCTTGAGGTGCTACTG-3′ and R: 5′-CTGACTTGACTTGGATTCTG-3′; the primers for GAPDH were F: 5′-AAGGTGAAGGTCGGAGTCAAC-3′ and R: 5′-GGGGTCATTGATGGCAACAATA-3′. Relative quantification of *mTOR* mRNA was calculated by using the 2-ΔΔCt method. Fold changes were normalized with respect to GAPDH, and each assay was done in triplicate.

#### Statistical analysis

Hardy-Weinberg equilibrium (HWE) was evaluated by the chi-square goodness of fit test to compare the observed genotype frequencies with the expected among the controls. Associations between genotypes and risk of gastric cancer were estimated by computing odds ratios (ORs) and 95% confidence intervals (CIs) from logistic regression analyses with adjustment for age and sex. Two-sided χ^2^ tests of statistical significance were performed by using SAS software (version 9.1.3; SAS Institute, Inc., Cary, NC, USA) and *P*<0.05 was considered statistical significance.

## Results

### Characteristics of the Study Subjects

The selected characteristics of these cases and controls are summarized in Table 1. The cases and controls appeared to be adequately matched on age (*P* = 0.424) and sex (*P* = 0.406). Among the 753 cases, tumor sites included 295 cardia cancer (39.2%) and 458 non-cardia cancer (60.8%). Patients with diffuse type (437, 58.0%) showed a slightly higher ratio than intestinal type (316, 42.0%). Based on the TNM classification of the American Joint Committee on Cancer (AJCC cancer staging manual, 6^th^ edition) [19], 17.3%, 17.3%, 50.6%, and 14.8% of patients had T1, T2, T3, and T4, respectively. Meanwhile, 60.6% of patients presented positive lymph node metastasis and 13.0% existed distant metastasis. According to these clinical characteristics, all patients were finally identified to stage I, II, III, and IV with 26.8%, 21.9%, 35.3%, and 16.0%, respectively.

**Table 1:** Selected characteristics between gastric cancer cases and healthy controls.

Variables	Cases (n = 753)		Controls (n = 854)		*P* a
	n	%	n	%	
Age (years)					
≤65	432	57.4	473	55.4	0.424
>65	321	42.6	381	44.6	
Sex					
Male	512	68.0	564	66.0	0.406
Female	241	32.0	290	34.0	
Tumor sites					
Cardia	295	39.2			
Non-cardia	458	60.8			
Histological types					
Diffuse	437	58.0			
Intestinal	316	42.0			
Depth of invasion					
T1	130	17.3			
T2	130	17.3			
T3	381	50.6			
T4	112	14.8			
Lymph node metastasis					
N0	297	39.4			
N1/N2/N3	456	60.6			
Distant metastasis					
M0	655	87.0			
M1	98	13.0			
TNM stages					
I	202	26.8			
II	165	21.9			
III	266	35.3			
IV	120	16.0			

a Two-sided χ^2^ test for selected variables between the cases and controls.

### The Overall Effects of rs2295080 Polymorphism on the Risk of GC

The genotype distributions and allele frequencies of rs2295080 are presented in Table 2. The genotype frequencies in the controls were in agreement with the HWE model (*P* = 0.569). As shown in Table 2, the genotype frequencies of rs2295080 were 64.0%, 32.7%, and 3.3% for the TT, TG, and GG genotypes among the cases, and 58.2%, 35.7%, and 6.1% among the controls, respectively. The difference between the cases and controls was statistically significant (*P = *0.008). Also, the G allele frequency was significantly lower among cases than controls (19.7% versus 23.9%, *P* = 0.003). In addition, the combined TG/GG genotype frequency was lower among cases than controls (36.0% versus 41.8%, *P* = 0.016). When taking TT genotype as reference, we found that the variant genotypes (TG and GG) were associated with a decreased risk of GC in a dose-response manner compared with the TT genotype (adjusted OR = 0.83, 95% CI = 0.67–1.02 for TG, and 0.49, 0.30–0.80 for GG; *P*_trend_ = 0.003). Similarly, we also observed that the combined TG/GG genotypes associated with a statistically significantly lower susceptibility to GC compared with the TT genotype (0.78, 0.64–0.96). Taken together, these data suggested that the *mTOR* rs2295080 G allele may be a putative protective allele.

**Table 2:** Distribution of genotypes of *mTOR* rs2295080 polymorphism between gastric cancer cases and healthy controls and the association with gastric cancer risk.

Genotypes	Cases (n = 753)		Controls (n = 854)		*P* a	Adjusted OR (95% CI)^b^
	n	%	n	%		
TT	482	64.0	497	58.2	**0.008**	1.00 (reference)
TG	246	32.7	305	35.7		0.83 (0.67–1.02)
GG	25	3.3	52	6.1		**0.49 (0.30–0.80)**
TG/GG	271	36.0	357	41.8	**0.016**	**0.78 (0.64–0.96)**
T allele	1210	80.3	1299	76.1		1.00 (reference)
G allele	296	19.7	409	23.9	**0.003**	**0.77 (0.65–0.92)**
*P*_trend_					**0.003**	

a Two-sided χ^2^ test for either genotype distributions or allele frequencies between cases and controls.

b Adjusted for age and sex in logistic regression model.

### Males were more Susceptible to Gastric Cancer with rs2295080 Polymorphism

As it is well-established, age and sex were important factors in tumor carcinogenesis including gastric cancer. According to the stratification analysis with age and sex factors, we found that significant association was observed in male group, rather than in female group (Table 3). In males, carrying rs2295080 G allele was a significantly decreased risk factor (OR = 0.72, 95% CI = 0.56–0.93 for TG/GG versus TT), comparing with carrying T allele. However, there was no statistical difference between young and old subjects.

**Table 3:** Stratified analyses of *mTOR* rs2295080 genotype frequencies in gastric cancer patients and healthy controls by age and sex.

Genotypes	Age				Sex				
	≤ 65 years		>65 years		Male		Female		
	n (cases/ controls)	Adjusted OR (95% CI)^a^	n (cases/ controls)	Adjusted OR (95% CI)^a^	n (cases/ controls)	Adjusted OR (95% CI)^b^	n (cases/ controls)	Adjusted OR (95% CI)^b^	
TT	273/272	1.00 (reference)	209/225	1.00 (reference)	331/321	1.00 (reference)	151/176	1.00 (reference)	
TG	142/171	0.84 (0.64–1.12)	104/134	0.89 (0.64–1.23)	165/205	0.79 (0.61–1.02)	81/100	1.05 (0.72–1.54)	
GG	17/30	0.56 (0.30–1.04)	8/22	0.43 (0.19–1.01)	16/38	**0.39 (0.21–0.73)**	9/14	0.92 (0.38–2.25)	
TG/GG	159/201	0.80 (0.61–1.05)	112/156	0.82 (0.60–1.13)	181/243	**0.72 (0.56–0.93)**	90/114	1.04 (0.72–1.50)	
T allele	688/715	1.00 (reference)	522/584	1.00 (reference)	827/847	1.00 (reference)	383/452	1.00 (reference)	
G allele	176/231	0.80 (0.64–1.00)	120/178	0.79 (0.61–1.04)	197/281	**0.72 (0.58–0.88)**	99/128	1.01 (0.74–1.38)	
Allele *P* c		0.051		0.094		**0.002**		0.933	
*P*_trend_		**0.040**		**0.032**		**0.002**		0.542	

a Adjusted for sex in logistic regression model.

b Adjusted for age in logistic regression model.

c Two-sided χ^2^ test for allele comparison.

### The Stratified Analysis of the Associations between rs2295080 Polymorphism and Clinical Variables of Gastric Cancer

We carried out the stratification analysis of rs2295080 polymorphism in all subjects by clinical features of GC. Among all independent variables, the protective effects were mainly observed in subgroups of patients with cardia gastric cancer (OR = 0.68, 95% CI = 0.52–0.91), intestinal type (0.73, 0.56–0.96), T3 depth invasion (0.78, 0.60–1.00), positive lymph node metastasis (0.79, 0.62–1.00), negative distant metastasis (0.76, 0.62–0.94), and localized stage (0.76, 0.59–0.98) (Table 4).

**Table 4:** The associations between *mTOR* rs22905080 polymorphism and clinical features of gastric cancer.

Variables	*mTOR* rs2295080				
	TT	TG/GG	*P* a	Adjusted OR (95% CI)^b^	*P* c
Controls (n = 854)	497 (58.2)	357 (41.8)	**0.016**	**0.78 (0.64–0.96)**	
Cases (n = 753)					
Tumor sites					
Cardia	198 (67.1)	97 (32.9)	**0.008**	**0.68 (0.52–0.91)**	0.234
Non-cardia	284 (62.0)	174 (38.0)	0.176	0.85 (0.67–1.08)	
Histological types					
Diffuse	275 (62.9)	162 (37.1)	0.104	0.82 (0.65–1.04)	0.524
Intestinal	207 (65.5)	109 (34.5)	**0.022**	**0.73 (0.56–0.96)**	
Depth of invasion					
T1	86 (66.2)	44 (33.8)	0.082	0.71 (0.48.1.04)	0.937
T2	82 (63.1)	48 (36.9)	0.329	0.83 (0.56–1.21)	
T3	244 (64.0)	137 (36.0)	**0.047**	**0.78 (0.60–1.00)**	
T4	70 (62.5)	42 (37.5)	0.364	0.83 (0.55–1.24)	
Lymph node metastasis					
N0	191 (64.3)	106 (35.7)	0.058	0.77 (0.58–1.01)	0.891
N1/N2/N3	291 (63.8)	165 (36.2)	**0.047**	**0.79 (0.62–1.00)**	
Distant metastasis					
M0	423 (64.6)	232 (35.4)	**0.011**	**0.76 (0.62–0.94)**	0.454
M1	59 (60.2)	39 (39.8)	0.672	0.91 (0.60–1.40)	
TNM stages					
Localized (I+II)	237 (64.6)	130 (35.4)	**0.033**	**0.76 (0.59–0.98)**	0.777
Advanced (III+IV)	245 (63.5)	141 (36.5)	0.077	0.80 (0.62–1.02)	

a Two-sided χ^2^ test for the frequency distributions of selected variables between cases and controls.

b Adjusted for age and sex in logistic regression model.

c Q-test for heterogeneity test.

### Effect of rs2295080 Polymorphism on Transcriptional Activity

**Figure 1:**
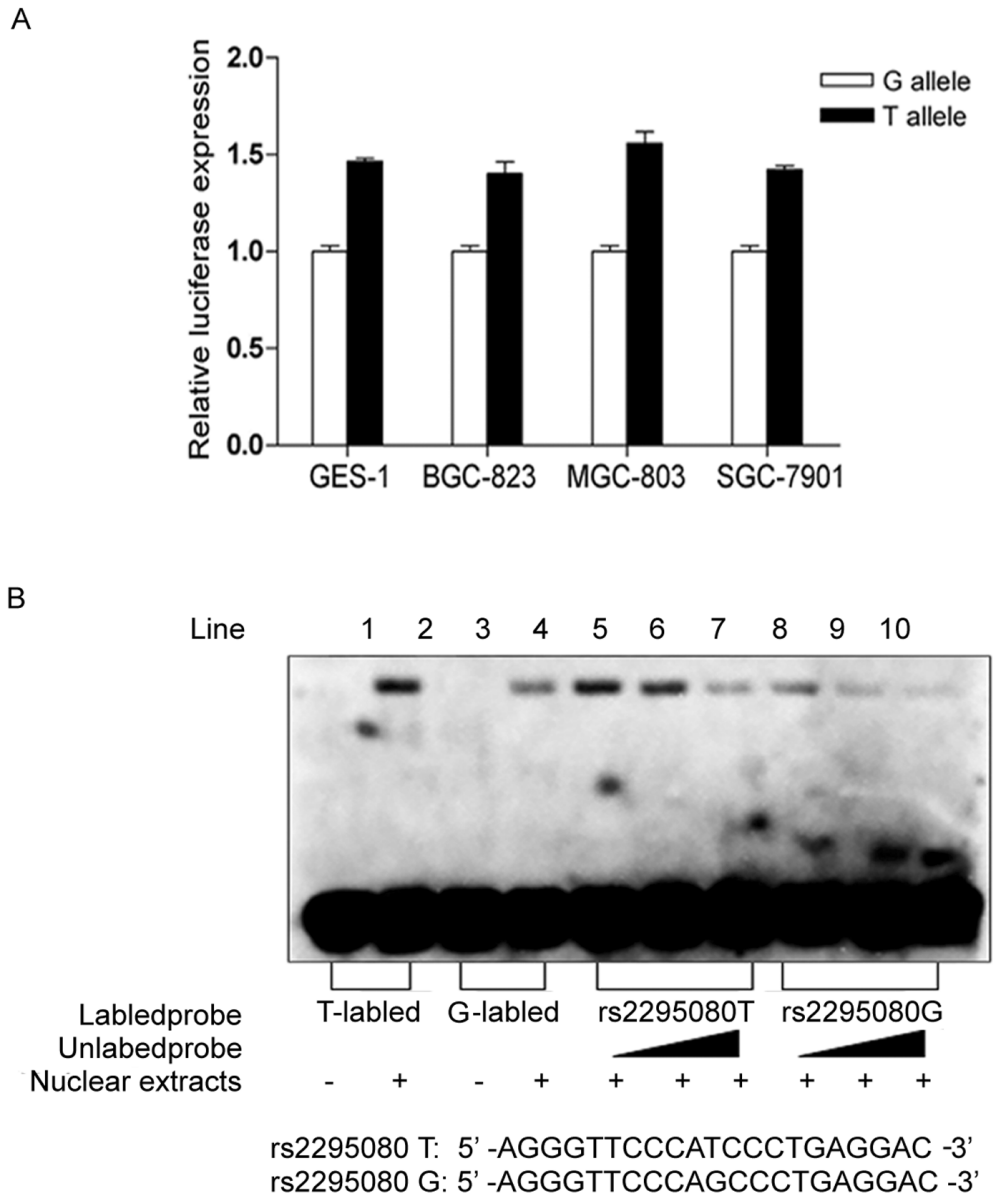
Functional analysis of rs2295080 polymorphism in *mTOR* promoter region. (A) Promoter activity of different alleles of *mTOR* rs2295080 polymorphism. T allele was described approximately 0.5-fold over G allele ability within luciferase assay of four sorts of gastric cell lines (GES-1, BGC-823, MGC-803 and SGC-7901). (B) Nuclear proteins binding activity of different alleles of *mTOR* rs2295080 polymorphism. Biotinylated probes (20 fmol) were incubated with 8 µg of nuclear extracts from SGC-7901 cells. In competition experiments, 10-, 100-, and 200-fold molar excess of unlabeled rs2295080 T/rs2295080 G probes were utilized to demonstrate the specificity of each binding reaction.

To gain an insight into the biological functional effect of rs2295080 polymorphism on *mTOR* transcription, GES-1, BGC823, MGC803, and SGC-7901 cells were transfected with different luciferase report plasmids, including wild type (T allele) and mutate type (G allele). As shown in Fig. 1A, we found that the transcription activity of T allele was higher than G allele with an approximately 1.5-fold in above four cell lines, suggesting that rs2295080 G allele worked as a defender for gastric cancer by reducing the transcription of *mTOR*.

### Nuclear Protein Binding Activity of Variants in rs2295080

Next, we performed an EMSA experiment to analyze the biological consequences of rs2295050 polymorphism in SGC-7901 cells. One strong band (protein complex) was shifted when the nuclear extracts were incubated with biotin-labeled rs2295080 T probe, whereas the weaker band was observed when the nuclear extracts were incubated with biotin-labeled with rs2295080 G probe. The shifted bands were significantly inhibited by a molar excess of an unlabeled rs2295080 T or rs2295080 G competitor in a dose-depend manner (Fig. 1B). These results indicated that rs2295080 G allele could decrease the nuclear protein binding activity to some extent.

### Association between *mTOR* rs2295080 Polymorphism and the Expression Levels of *mTOR* mRNA

**Figure 2:**
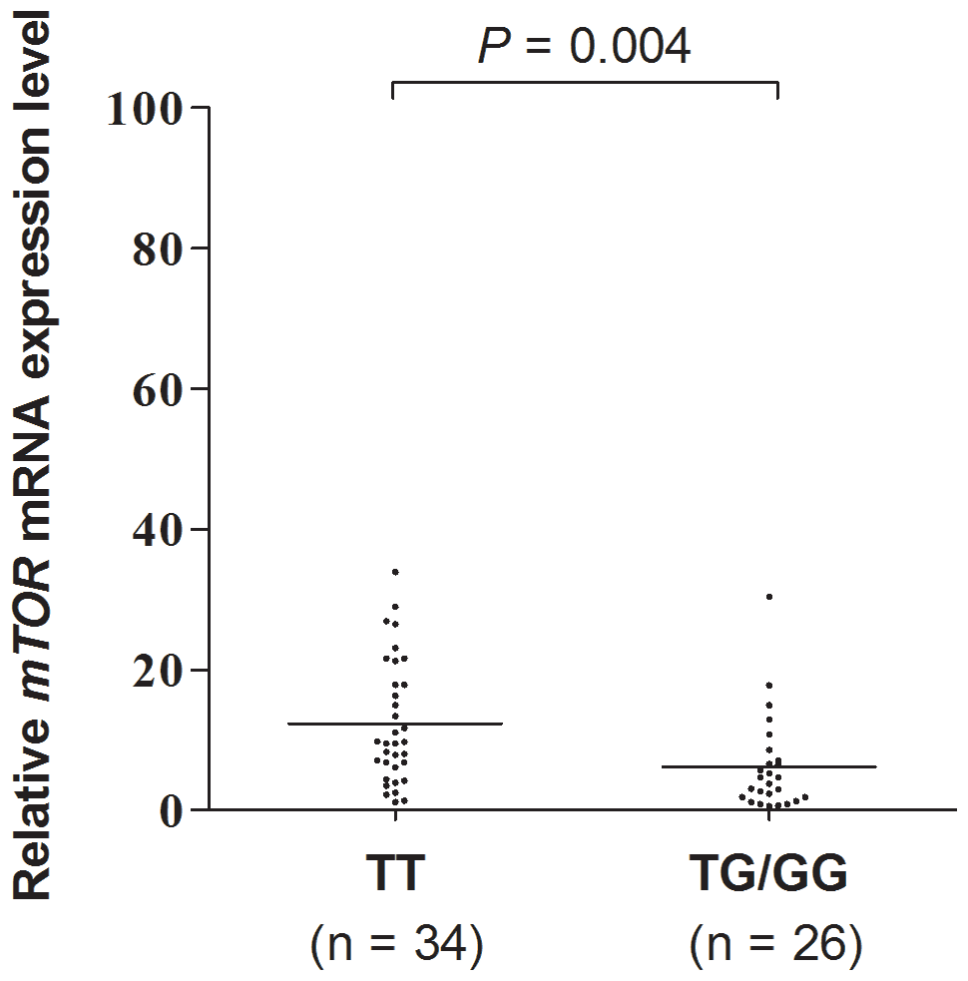
Association between rs2295080 polymorphism in *mTOR* promoter region and *mTOR* mRNA levels in gastric cancer cases (n = 60). TT versus TG/GG genotypes, *P* = 0.004.

Sixty-three gastric cancer tissues with different genotypes of *mTOR* rs2295080 were available in our present study. Because of the low frequency of GG genotype, we added it into the samples with TG genotype for analysis. As shown in Fig. 2, the expression levels of *mTOR* mRNA was dramatically higher in individuals with TT genotype than in those with TG or GG genotype (Mean ± SEM of TT versus TG/GG: 12.35±1.51 versus 6.17±1.32, *P* = 0.004).

## Discussion

In the present study, we demonstrated that the G allele of *mTOR* promoter polymorphism rs2295080 was associated with a significantly decreased risk of GC. We found that the T to G change in rs2295080 substantially altered transcriptional activity of *mTOR* gene via influencing the binding of some transcriptional factor. We also observed that *mTOR* rs2295080 T allele was associated with higher *mTOR* mRNA expression levels *in vivo*.

Mammalian target of rapamycin (mTOR), also known as FRAP1, is one necessary member of PI3K/Akt/mTOR pathway and central to metabolic signaling [20], [21]. It can be activated by insulin, insulin-like growth factors and other growth factors, and inactivated during cellular starvation. mTOR exists two different kinds of complex, named mTORC1 and mTORC2, respectively [22]. The mTORC1 complex phosphorylates its downstream effecter p70S6k, which could induce the degradation of insulin receptor substrate and further increase the insulin-driven Akt activity. The mTORC2 complex phosphorylates the C-terminus of Akt at ser473, which could lead to the entire activation of Akt [9]. As a therapeutic target in other cancers [4], [8], [23]–[27], mTOR could also emerged as a potential target for treatment of gastric cancer, spontaneously. Sirolimus [28] and everolimus [29], the mTOR inhibitors, were identified to result in G1 cell cycle arrest and inhibited the proliferation of gastric cell lines. Recently, a novel potential *mTOR* promoter polymorphism rs2295080 has been described in several studies [30]–[32]. However, insufficient functional studies were performed to evaluate the role of this genetic variant in regulating the *mTOR* expression. In GC, our findings provided the evidence that rs2295080 T allele could enhance the transcription activity of *mTOR* to some extent in GES-1 cell line after transfected *in vitro*. And further luciferase assay in three gastric cancer cell lines (i.e., BGC-823, MGC-803, and SGC-7901) further confirmed this effect of rs2295080 polymorphism. These results in our present study were in accordance with the outcomes in renal cancer cell line and cervix cancer cell line [32]. In EMSA assay, rs2295080 was predicted to locate on the potential binding site, whose polymorphic variants could influence the recruit of transcription factors (using the p-MATCH program, which uses binding sites in TRANSFAC; www.gene-regulation.com). Taken these results together, it is plausible that *mTOR* rs2295080 polymorphism could influence the expression level of *mTOR* and individual susceptibility to various cancers.

The SNP rs2295080, as well as another intron SNP rs11121704, was first reported by Hildebrandt *et al.* to locate in *mTOR* potential promoter region [13]. As described in esophageal cancer, rs2295080 seemed no association or function with survival after chemoradiotherapy and surgery. But until now, several studies have identified the effect of this polymorphism in *mTOR* for cancer risk [30]–[32], all of which considered rs2295080 T allele as a risk factor. In the current study, the association between rs2295080 T allele and GC risk was consistent with the results of previous three studies [30]–[32]. In addition, our results also suggested that this polymorphism might be a biological crux in the development of GC. Because of different populations and different mechanisms among occurrence, development, and survival, we speculated the ethnicity differences and mechanistic distinctions might explain these discordances.

Another novel result comes from the association between genders and *mTOR* rs2295080 polymorphism in stratified analysis. This rs2295080 polymorphism revealed more strong significance in men than women. Additionally, Hartgrink *et al*. (2009) has reported the ratio of men to women infected by gastric cancer is about 2∶1 per annum [33]. Further explanation for connections of these results needs to be sought, and different sorts of hormones expression in males and females become the best consideration, rationally. In a study of breast cancer, Galoian *et al.* has given the data that prolinerich polypeptide-1 exerts antiproliferative effect via inhibiting mTOR kinase activity in ER-negative MDA-231, but no inhibitory effect exists in luminal T47-D cell which performs as an ER-positive cell line [34]. However, for studies of androgen, it was believed that androgen might stimulate mTOR activity in PTEN-deficient prostate cancer cells. A recent study on androgen receptor improved the role of androgen in up-regulating mTORC2 activity [35]. It is likely that androgen, instead of estrogen, may increase the *mTOR* expression; androgen may lead to the high incidence of gastric cancer in men and the strong significance of *mTOR* rs2295080 in male group in our study.

Some limitations of our present study should be pointed out as follows. First, rs2295080 was not the only polymorphism significantly existing on *mTOR*, and the importance of combining SNPs was neglected in our study. Second, as crucial factors in gastric carcinogenesis, the lacks of *Helicobacter pylori* information, smoking information, and drinking information were also insufficient in our study, it is untoward for us for further investigation of the effects of this polymorphism. In addition, because of the relative small sample size in our study, no significant results seemed to be found in particular strata (age subgroups, female subgroup, and other clinico-pathological subgroups) of stratified analyses (Table S3 and S4). Further larger study with more information was expected to verify our findings.

In conclusion, our study illuminated the *mTOR* rs2295080 locating in the promoter region of *mTOR* gene was significantly associated with risk of gastric cancer in a Chinese population.

## Supporting Information

Table S1 Primers and probes used for genotyping.
(DOC)

Table S2 Primers for construction of plasmids.
(DOC)

Table S3 Interaction analyses of *mTOR* rs2295080 polymorphism and age or sex status in case-control study.
(DOC)

Table S4 Interaction analyses of *mTOR* rs2295080 polymorphism and age/sex or clinical characters in case-only study.
(DOC)
