# Supplementary material for: A Polymorphism (rs2295080) in mTOR Promoter Region and Its Association with Gastric Cancer in a Chinese Population
Source: PLoS One. 2013 Mar 29;8(3):e60080. doi: 10.1371/journal.pone.0060080 (PMC3612103; doi:10.1371/journal.pone.0060080)
Supplement: Table S1 — Primers and probes used for genotyping. (DOC) [file pone.0060080.s001.doc]

**Table S1.** Primers and probes used for genotyping.

| SNP | Primers and probes | Sequence (5’-3’) |
| --- | --- | --- |
| rs2295080 | F | CTTCCCCGCTGTCCTCTAAG |
|  | R | CCATCTTCTCCCTATACCTGTCG |
|  | G | HEX-CTCAGGGCTGGGAA-MGB |
|  | T | FAM-TCAGGGATGGGAAC-MGB |
