# Supplementary material for: A Polymorphism (rs2295080) in mTOR Promoter Region and Its Association with Gastric Cancer in a Chinese Population
Source: PLoS One. 2013 Mar 29;8(3):e60080. doi: 10.1371/journal.pone.0060080 (PMC3612103; doi:10.1371/journal.pone.0060080)
Supplement: Table S2 — Primers for construction of plasmids. (DOC) [file pone.0060080.s002.doc]

**Table S2. Primers for construction of plasmids**.

| Primers and probes | Sequence (5’-3’) |
| --- | --- |
| Forward | ACTTAGAGCTCAAACAGGGATGGGGCTGGGGGAGAGG​GA |
| Reverse | ACTTAAGATCTCGAAACGTCTTTTGATGCAGTAATTC​CT |
