# Supplementary material for: A Polymorphism (rs2295080) in mTOR Promoter Region and Its Association with Gastric Cancer in a Chinese Population
Source: PLoS One. 2013 Mar 29;8(3):e60080. doi: 10.1371/journal.pone.0060080 (PMC3612103; doi:10.1371/journal.pone.0060080)
Supplement: Table S3 — Interaction analyses of mTOR rs2295080 polymorphism and age or sex status in case-control study. (DOC) [file pone.0060080.s003.doc]

**Table S3.** Interaction analyses of *mTOR* rs2295080 polymorphism and age or sex status in case-control study.

| Age or sex status | Genotypes | Cases (n = 753) | |  | Controls (n = 854) | | *P*^a^ | Adjusted OR (95% CI)^b^ |
| --- | --- | --- | --- | --- | --- | --- | --- | --- |
|  |  | n | % |  | n | % |  |  |
| Age > 65 years | TG/GG | 112 | 14.9 |  | 156 | 18.3 |  | 1.00 (reference) |
| > 65 years | TT | 209 | 27.8 |  | 225 | 26.4 | 0.091 | 1.30 (0.96-1.77) |
| ≤ 65 years | TG/GG | 159 | 21.1 |  | 201 | 23.5 | 0.452 | 1.13 (0.82-1.56) |
| ≤ 65 years | TT | 273 | 36.2 |  | 272 | 31.8 | **0.018** | **1.43 (1.06-1.93)** |
| *P*_interaction_^c^ |  |  |  |  |  |  | 0.679 |  |
| Sex: Male | TG/GG | 181 | 24.0 |  | 243 | 28.4 |  | 1.00 (reference) |
| Male | TT | 331 | 44.0 |  | 321 | 37.6 | **0.010** | **1.38 (1.08-1.77)** |
| Female | TG/GG | 90 | 12.0 |  | 114 | 13.4 | 0.857 | 1.03 (0.73-1.45) |
| Female | TT | 151 | 20.0 |  | 176 | 20.6 | 0.407 | 1.13 (0.84-1.52) |
| *P*_interaction_^c^ |  |  |  |  |  |  | 0.269 |  |

^a^ Two-sided χ^2^ test for the frequency distributions between cases and controls.

^b^ Adjusted for sex (for age-gene interaction) or age (for sex-gene interaction) in logistic regression model.

^c^ Multiplicative model was used for interaction analyses.
