# Supplementary material for: A Polymorphism (rs2295080) in mTOR Promoter Region and Its Association with Gastric Cancer in a Chinese Population
Source: PLoS One. 2013 Mar 29;8(3):e60080. doi: 10.1371/journal.pone.0060080 (PMC3612103; doi:10.1371/journal.pone.0060080)
Supplement: Table S4 — Interaction analyses of mTOR rs2295080 polymorphism and age/sex or clinical characters in case-only study. (DOC) [file pone.0060080.s004.doc]

**Table S4.** Interaction analyses of *mTOR* rs2295080 polymorphism and age/sex or clinical characters in case-only study.

| Variables | *mTOR* rs2295080 | | | |
| --- | --- | --- | --- | --- |
|  | TT | TG/GG | *P* _interaction_^a^ | Adjusted OR  (95% CI)^b^ |
| Cases (n = 753) |  |  |  |  |
| Age |  |  | 0.577 | 0.92 (0.68-1.24) |
| ≤ 65 years | 273 (63.2) | 159 (36.8) |  |  |
| > 65 years | 209 (65.1) | 112 (34.9) |  |  |
| Sex |  |  | 0.584 | 1.09 (0.80-1.50) |
| Male | 331 (64.6) | 181 (35.4) |  |  |
| Female | 151 (62.7) | 90 (37.3) |  |  |
| Sites |  |  | 0.159 | 1.25 (0.92-1.70) |
| Cardia | 198 (67.1) | 97 (32.9) |  |  |
| Non-cardia | 284 (62.0) | 174 (38.0) |  |  |
| Histological types |  |  | 0.528 | 0.91 (0.67-1.23) |
| Diffuse | 275 (62.9) | 162 (37.1) |  |  |
| Intestinal | 207 (65.5) | 109 (34.5) |  |  |
| Depth of invasion |  |  | 0.601 | 1.04 (0.89-1.22) |
| T1 | 86 (66.2) | 44 (33.8) |  |  |
| T2 | 82 (63.1) | 48 (36.9) |  |  |
| T3 | 244 (64.0) | 137 (36.0) |  |  |
| T4 | 70 (62.5) | 42 (37.5) |  |  |
| Lymph node metastasis |  |  | 0.891 | 1.02 (0.75-1.39) |
| N0 | 191 (64.3) | 106 (35.7) |  |  |
| N1/N2/N3 | 291 (63.8) | 165 (36.2) |  |  |
| Distant metastasis |  |  | 0.391 | 1.21 (0.78-1.87) |
| M0 | 423 (64.6) | 232 (35.4) |  |  |
| M1 | 59 (60.2) | 39 (39.8) |  |  |
| Stages |  |  | 0.756 | 1.05 (0.78-1.41) |
| Localized (I/II) | 237 (64.6) | 130 (35.4) |  |  |
| Advanced (III/IV) | 245 (63.5) | 141 (36.5) |  |  |

^a^ Multiplicative model was used for interaction analyses.

^b^ Adjusted for age and sex in logistic regression model.
